# Exacerbation of Autoimmune Bullous Diseases After Severe Acute Respiratory Syndrome Coronavirus 2 Vaccination: Is There Any Association?

**DOI:** 10.3389/fmed.2022.957169

**Published:** 2022-07-19

**Authors:** Nika Kianfar, Shayan Dasdar, Ali Salehi Farid, Kamran Balighi, Hamidreza Mahmoudi, Maryam Daneshpazhooh

**Affiliations:** Autoimmune Bullous Diseases Research Center, Tehran University of Medical Sciences, Tehran, Iran

**Keywords:** SARS-CoV-2 vaccination, COVID-19, vaccine, disease activity, autoimmune bullous dermatoses, disease exacerbation

## Abstract

**Background and Aim:**

There have been concerns regarding the potential exacerbation of autoimmune bullous diseases (AIBDs) following vaccination against COVID-19 during the pandemic. In the current study, vaccine safety was evaluated in patients with AIBDs.

**Methods:**

In this study, patients with AIBDs were contacted via face-to-face visits or phone calls. Patient demographics, vaccine-related information, pre- and post-vaccine disease status, and complications were recorded. The exacerbation was considered either relapse in the remission/controlled phase of the disease or disease worsening in the active phase. The univariate and multivariate logistic regression tests were employed to determine the potential risk factors of disease exacerbation.

**Results:**

Of the patients contacted, 446 (74.3%) reported receiving at least one dose of vaccine injection (54.7% female). Post-vaccine exacerbation occurred in 66 (14.8%) patients. Besides, there were 5 (1.1%) patients with AIBD diagnosis after vaccination. According to the analysis, for every three patients who received vaccines during the active phase of the disease one experienced disease exacerbation. The rate of disease exacerbation increased by three percent with every passing month from the last rituximab infusion. Active disease in the past year was another risk factor with a number needed to harm of 10.

**Conclusion:**

Risk of AIBD exacerbation after the COVID-19 vaccine is not high enough to prevent vaccination. This unwanted side effect, can be reduced if the disease is controlled at the time of vaccination.

## Introduction

Autoimmune bullous diseases (AIBDs) are a group of blistering dermatoses of the skin and mucosa. Vaccinations have always been a challenging issue for patients with AIBDs and their physicians for the possible risk of disease exacerbation (1).

The severe acute respiratory syndrome coronavirus 2 (SARS-CoV-2) outbreak began in Wuhan, China, in December 2019 and was declared a global pandemic by World Health Organization in March 2020 (2). Patients with AIBDs may be at increased risk of severe complications of coronavirus disease 2019 (COVID-19), and they are in substantial need to undergo vaccination (3–5). However, those with an underlying disease like AIBDs were excluded from vaccine clinical trials, and little is known concerning the safety profile of SARS-CoV-2 vaccines in this population.

Some studies have reported cases of exacerbation or new-onset AIBDs post-SARS-CoV-2 vaccination (6–9), yet it has not been evaluated in larger series. Herein, we sought to determine whether SARS-CoV-2 vaccination might affect the natural course of AIBDs and find the potential risk factors of disease exacerbation.

## Materials and Methods

### Study Design and Participants

This cross-sectional study was performed at the AIBD clinic of Razi Skin hospital, Tehran, Iran, for 3 months between September 10 and December 10, 2021. To evaluate the SARS-CoV-2 vaccine outcome, AIBD patients were contacted, and those who had received at least one vaccine shot were enrolled.

Informed consent was obtained from each patient verbally. The study was conducted according to the Helsinki Declaration, and ethical approval was obtained from the Tehran University of Medical Sciences Ethics committee (IR.TUMS.MEDICINE. REC.1400.911). The patients were interviewed through face-to-face visits or phone calls. Demographic data, vaccine-related information, pre- and post-vaccine disease status, and vaccine-induced complications were self-reported by the patients and completed by referring to their medical records. If the patients had received only the first dose vaccination, they were contacted again by the follow-up phone call 1 month after the scheduled vaccination time.

To assess the post-vaccination disease status, an in-person visit was set for a thorough examination of the patients who reported new lesions after vaccination on the telephone interviews. Moreover, if this had occurred in the past, patients’ medical records were used to extract the necessary information. It is noteworthy that patients whose information was not complete and accurate were not included. The SARS-CoV-2 diagnosis was based on a positive polymerase chain reaction (PCR) test result or lung involvement on chest computed tomography (CT) scan congruent with SARS-CoV-2 (2). The patients were enrolled based on the following inclusion and exclusion criteria:

Inclusion criteria:

• Definite diagnosis of AIBDs
• Patient’s willingness to participate
• Receiving at least one vaccine shot

Exclusion criteria:

• Patient’s inaccessibility if an in-person examination was needed
• Incomplete or imprecise data

### Definition of Exacerbation

In patients with active disease, a 10-point increase in Pemphigus Disease Area Index (PDAI)/Bullous Pemphigoid Disease Area Index (BPDAI)/Mucous Membrane Pemphigoid Disease Area Index (MMPDAI) was defined as disease worsening (10). For linear IgA disease and epidermolysis bullosa acquisita, BPDAI and MMPDAI scorings were employed, respectively. Relapse or newly diagnosed uncontrolled cases were considered to be in the active phase of the disease.

Relapse in patients with disease remission corresponded to the appearance of ≥ 3 new lesions in a month that did not heal spontaneously within 1 week, and in those with controlled disease corresponded to extension of established lesions (11). To determine the severity of relapses, the emergence of ≥ 20 lesions on ≥ 3 body sectors was defined as major relapse, and the appearance of < 20 lesions on < 3 body sectors as minor relapse (12). New lesions that did not comply with any of these criteria were considered as transient lesions. According to a previous study, the exacerbation must be occurred within 2 weeks of the vaccines to be considered as vaccine-associated (13). In summary, the criteria for defining the vaccine-associated exacerbations were as follows:

• A 10-point increase in PDAI/BPDAI/MMPDAI in the active phase of the disease
• ≥3 new lesions or extension of established lesions in the remission/controlled phase of the disease
• Occurrence within 2 weeks of the vaccine

### Statistical Analysis

Absolute number and percentage were employed for reporting qualitative variables. The quantitative variables were presented as mean with standard deviation (SD) for normal distributed variables and median with interquartile range (IQR) for non-normal ones. Logistic regression analysis was employed to predict dependent variable of post-vaccination disease exacerbation. Odds ratio (OR) with 95% confidence interval (95% CI) were measured for demographic and clinical data of the patients. Factors that demonstrated statistical significance (*p* < 0.05) based on univariate analysis and had no obvious colinearity with other variables were incorporated into multivariate analysis. Number needed to harm was measured for detected categorical risk factors reciprocating the absolute risk reduction (difference of event rate between two groups). All analyses were performed using SPSS (version 24; IBM, New York, United States), and statistical significance was defined as *P* < 0.05.

## Results

**FIGURE 1:**
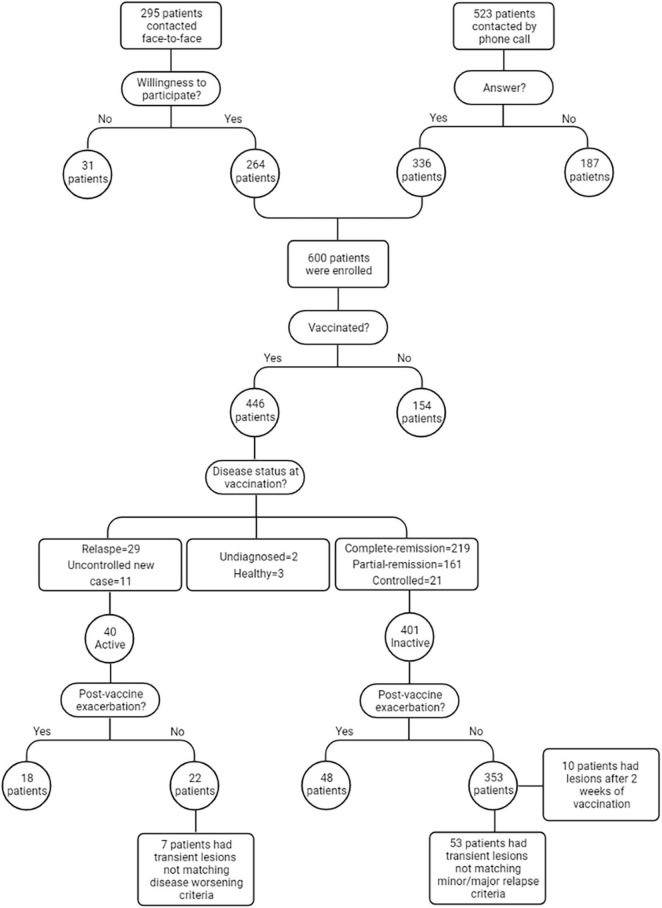
Methodology of the study.

Six hundred patients with AIBD diagnosis were contacted. The flow diagram of patients’ enrollment is illustrated in Figure 1. Of the patients, 446 (74.3%) reported receiving at least one dose of vaccine injection (54.7% female). Supplementary Figure 1 compares the first dose SARS-CoV-2 vaccination rates between the present study and the country’s general population. Vaccines used in patients were Sinopharm (BBIBP-CorV, inactivated virus vaccine) in 379 (85.9%), AstraZeneca (ChAdOx1 nCoV-19, viral vector vaccine) in 36 (8.0%), COVIran Barekat (BIV1-CovIran, inactivated virus vaccine) in 22 (4.9%), and others in 9 (2.0%). The second vaccine shot was received by 404 (67.3%) patients before the defined cut-off date (55.9% female). The reasons for not completing vaccinations in these patients were as follows: not reaching the time in 11 (26.2%) patients, uncontrolled disease/relapse in 9 (21.4%), COVID-19/flu in 9 (21.4%), rituximab administration in 1 (2.4%), and non-medical reasons in 12 (28.5%).

The mean age of the 446 vaccinated patients was 50.2 ± 12.5 years old. The types of AIBDs were pemphigus vulgaris in 361 (80.9%) patients, pemphigus foliaceus in 38 (8.5%), bullous pemphigoid in 29 (6.5%), mucous membrane pemphigoid in 13 (2.9%), linear IgA disease in 2 (0.4%), epidermolysis bullosa acquisita in 2 (0.4%), and paraneoplastic pemphigus in 1 (0.2%). Of the patients, 334 (74.9%) were on systematic medications, mostly prednisolone (73.1%) at the time of vaccination. Twenty-nine patients (6.5%) were above minimal therapy (>10 mg/d prednisolone or equivalent). There were 184 (41.3%) patients who had a previous history of COVID-19 affection, of whom 118 (26.5%) had confirmed diagnosis of COVID-19 and 39 (8.7%) were hospitalized. Among these infections, 31 (6.9%) occurred within 3 months of the first vaccine injection, and the others were before that. The detailed data of clinical characteristics and vaccine-related information of the patients are depicted in Table 1.

**TABLE 1:** Clinical characteristics and vaccine-related data of patients with AIBDs.

		Total population = 446	
Age (years old), mean ± SD		50.2 ± 12.5	
Types of AIBDs	Pemphigus vulgaris	361 (80.9%)	
	Pemphigus foliaceus	38 (8.5%)	
	Bullous pemphigoid	29 (6.5%)	
	Mucous membrane pemphigoid	13 (2.9%)	
	Linear IgA disease	2 (0.4%)	
	Epidermolysis bullosa acquisita	2 (0.4%)	
	Paraneoplastic pemphigus	1 (0.2%)	
Disease duration until vaccination (months), median (IQR)		56.3 (31–109)	
Previous COVID-19 experience, n (%)	COVID-19 symptoms	184 (41.3%)	
	Diagnosed with COVID-19	118 (26.5%)	
	Hospitalized COVID-19	39 (8.7%)	
Bullous disease status at vaccination, n (%)	CR on minimal or off-therapy	219 (49.1%)	
	PR on minimal or off-therapy	161 (36.1%)	
	Controlled	21 (4.7%)	
	Relapse	29 (6.5%)	
	Uncontrolled new cases	11 (2.5%)	
	Undiagnosed or healthy	5 (1.1%)	
Duration from last RTX infusion (months), median (IQR)		21.4 (10.3–33.3)	
Disease severity at vaccination, median (range)	PDAI score	0 (0–22)	
	BPDAI score	0 (0–20)	
	MMPDAI	0 (0–17)	
Medication at vaccination, n (%)	Prednisolone, mg [dose, median (IQR)]	326 (73.1%), [5 (3.75–8.75)]	
	Mycophenolate mofetil	17 (3.8%)	
	Topical	40 (9.0%)	
	Other systemic	18 (4.0%)	
	No medication	112 (25.1%)	
Other risk factors of relapse, n (%)	Comorbidity^a^	234 (52.5%)	
	Major stress	65 (14.6%)	
	Medication reduction/cessation	11 (2.5%)	
	Infection^b^	9 (2.0%)	

**Vaccination^c^**		**First dose vaccination**	**Second dose vaccination**

First vaccine type, n (%)	Sinopharm	379 (85.9%)	348 (86.1%)
	AstraZeneca	36 (8.0%)	28 (6.9%)
	COVIran Barekat	22 (4.9%)	20 (5.0%)
	Other	9 (2.0%)	8 (2.0%)
Vaccine side effects, n (%)	Pain at the injection site	129 (28.9%)	92 (22.8%)
	Fatigue	82 (18.3%)	57 (14.1%)
	Fever/chill, flu-like symptoms	56 (12.5%)	32 (7.9%)
	Headache	44 (9.9%)	33 (8.2%)
	Dizziness	10 (2.2%)	4 (1.0%)
	Myalgia	38 (8.5%)	25 (6.2%)
	Gastrointestinal symptoms	9 (2.0%)	10 (2.5%)
	Cutaneous reactions	2 (0.4%)	1 (0.2%)
	Anaphylactic shock	0 (0%)	0 (0%)
COVID infection after vaccination		10 (2.2%)	7 (1.7%)
Interventions for vaccine side effects, n (%)	No medication	109 (49.3%)	75 (49.3%)
	Over the counter drugs	110 (49.8%)	75 (49.3%)
	Hospitalization	2 (0.9%)	2 (1.3%)
Disease exacerbation after vaccination^d^, n (%)	Disease worsening	12 (2.7%)	23 (5.7%)
	Minor relapse	21 (4.7%)	13 (3.2%)
	Major relapse	9 (2.0%)	5 (1.2%)
	Total^e^	42 (9.4%)	41 (10.1%)
New diagnosis AIBD after vaccination, n (%)		3 (0.7%)	2 (0.5%)
Interval of vaccination to disease exacerbation (days), median (IQR)		7 (3.7–12.5)	7 (3–10)
Medication taken for post-vaccination disease activity, n (%)	No altered medication	6 (14.3%)	4 (9.7%)
	Topical	8 (19.0%)	5 (12.1%)
	Increase medication dosage	34 (80.9%)	35 (85.4%)
	New medication (other than RTX)	4 (9.5%)	1 (2.4%)
	RTX	5 (11.9%)	19 (46.4%)^f^

*^a^Recorded comorbidities were as follows: hyperlipidemia = 104, hypertension = 95, diabetes = 95, hypothyroidism = 40, cardiovascular diseases = 43, pulmonary diseases = 9, hepatic diseases = 8, renal diseases = 6, other autoimmune diseases = 5, hyperthyroidism = 4, and cancer = 3.*

*^b^Recorded infections were as follows: five COVID-19, two urinary tract infections, one pneumonia, and one herpes zoster.*

*^c^Sinopharm, BBIBP-CorV; AstraZeneca, ChAdOx1 nCoV-19; COVIran Barekat, BIV1-CovIran.*

*^d^Disease exacerbation after vaccination was categorized as disease worsening (10-point increase in PDAI/BPDAI/MMPDAI), minor relapse (< 20 lesions on < 3 body sectors), and major relapse (≥ 20 lesions on ≥ 3 body sectors).*

*^e^Seventeen patients with disease exacerbation after first vaccine shot experienced disease worsening after second vaccine as well. Nine patients with minor relapse, three patients with major relapse, and five patients with disease worsening after the first vaccine experienced disease worsening after the second dose.*

*^f^Nine patients were RTX candidates prior to vaccination due to uncontrolled disease. AIBDs, autoimmune bullous diseases; SD, standard deviation; IQR, interquartile range; CR, complete remission; PR, partial remission; PDAI, Pemphigus Disease Area Index; RTX, rituximab; BPDAI, Bullous Pemphigoid Disease Area Index; MMPDAI, Mucous Membrane Pemphigoid Disease Area Index.*

The three most common vaccine side effects were pain at the injection site in 221 (26%) vaccine shots, 139 (16.3%) fatigue, and 88 (10.3%) flu-like symptoms. Within 1 month of vaccinations, 17 (3.8%) patients were diagnosed as COVID-19. Of them, 14 (82.4%) were treated as outpatients, and the other 3 (17.6%) were administered remdesivir. A 56-year-old man who developed COVID-19 after the first vaccine dose, in the follow-up phone call, was reported to have passed away after hospitalization due to that infection. He was a known case of pemphigus vulgaris for 2 years with hypertension and diabetes. He was in partial remission on minimal therapy (prednisolone 5 mg) and received the first vaccine dose of Sinopharm (BBIBP-CorV); no vaccine-related side effects were reported, and the second dose was postponed for COVID-19 symptoms.

**FIGURE 2:**
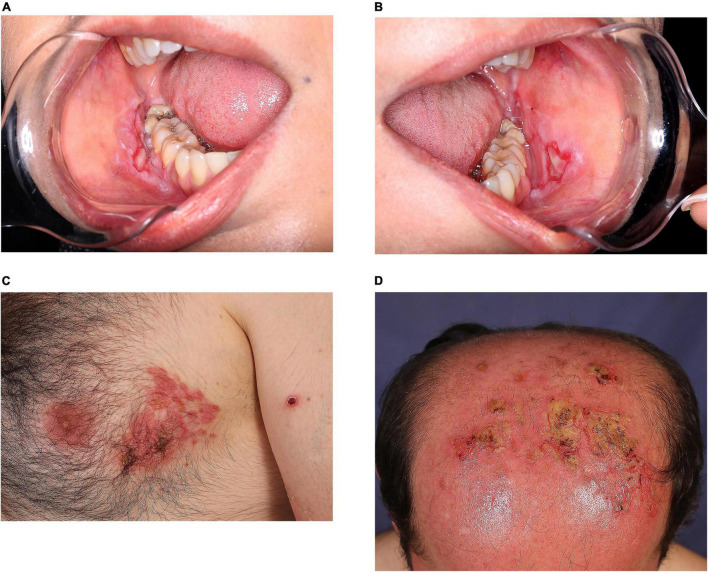
Clinical appearance of pemphigus vulgaris exacerbation in two patients vaccinated against SARS-CoV-2: a 39-year-old woman who experienced disease exacerbation 17 days after the first dose of Sinopharm vaccine **(A,B)**; a 46-year-old man who experienced disease exacerbation 7 days after the second dose of COVIran Barekat vaccine **(C,D)**.

A total of 66 (14.8%) patients experienced post-vaccine disease exacerbation. Of those 401 patients who were either in remission or were controlled, 34 (8.5%) patients experienced minor, and 14 (3.5%) experienced major relapses. Among 40 patients who received the vaccine in the active phase of the disease, 18 (45%) patients reported disease worsening post-vaccination. There were also five patients who were diagnosed as AIBDs after the vaccination. Two of them reported occasional lesions before the vaccine, and the other three denied any history of skin diseases and were otherwise healthy. Of the 404 fully vaccinated patients, only 17 (4.2%) developed disease exacerbation after both vaccine shots. Figure 2 demonstrates the two patients who reported disease exacerbation after the SARS-CoV-2 vaccination.

According to multivariate analysis, every passing month from the last rituximab infusion increased the rate of disease exacerbation by 3 percent (95% CI, 1.01–1.05) (*p* = 0.03). Vaccination in those with a history of active disease in the past year had an OR of 2.11 (95% CI, 1.49–6.49) (*p* = 0.009) for exacerbation, with a number needed to harm of 10. Moreover, vaccine injection in the active phase of the disease escalated the risk of exacerbation with an OR of 8.9 (95% CI, 3.6–22.0) (*p* < 0.001), corresponding to the number needed to harm of 3; suggesting that for every three patients who receive the vaccine at the active phase of the disease one would experience disease exacerbation.

There was no significant difference in the exacerbation rate of AIBD subtypes (*p* > 0.05), as the recorded rates were 16.5% in pemphigus vulgaris, 10.5% in pemphigus foliaceus, 3.8% in bullous pemphigoid, 23.1% in mucous membrane pemphigoid, and none in the others. Due to the importance of disease exacerbation in AIBD subtypes, the detailed information of each is presented in Supplementary Table 1.

Other factors, including age, sex, vaccine type, disease duration, prednisolone dosage, previous COVID-19 infection, and having risk factors of relapse (comorbidity, major stress, medication reduction/cessation, infection), had no significant effect on post-vaccination disease exacerbation (Table 2).

**TABLE 2:** Univariate and multivariate analysis for the probable factors of post-vaccination disease exacerbation in patients with AIBDs.

		Univariate		Multivariate	
		OR (95% CI)	*P*-value	OR (95% CI)	*P*-value
Age, years old		0.998 (0.977–1.019)	0.869		
Sex (female vs. male)		1.017 (0.601–1.719)	0.951		
Types of AIBDs (vs. pemphigus vulgaris)	Pemphigus foliaceus	0.611 (0.209–1.786)	0.368		
	Bullous pemphigoid	0.208 (0.028–1.562)	0.127		
	Mucous membrane pemphigoid	1.557 (0.416–5.831)	0.511		
Disease duration until vaccination, months		1.000 (0.997–1.004)	0.813		
Active disease in the past year		**2.025 (1.192–3.438)**	**0.009**	**3.111 (1.490–6.496)**	**0.003**
Previous COVID-19 experience		0.961 (0.565–1.636)	0.884		
Duration from last RTX infusion, months		**1.016 (1.002–1.031)**	**0.03**	**1.028 (1.011–1.045)**	**0.001**
Risk factors of relapse^a^		0.640 (0.378–1.083)	0.097		
Bullous disease status at vaccination (active^b^ vs. inactive^c^)		**6.017 (3.012–12.021)**	**<0.001**	**8.904 (3.600–22.022)**	**<0.001**
Prednisolone dosage at vaccination^d^, mg		**1.064 (1.018–1.112)**	**0.006**		
Vaccine type^e^ (vs. Sinopharm)	AstraZenca	1.335 (0.527–3.381)	0.542		
	COVIran Barekat	2.337 (0.874–6.244)	0.091		
	Others	1.780 (0.360–8.804)	0.479		

*^a^Comorbidity, major stress, medication reduction/cessation, infection.*

*^b^Active: relapse or newly diagnosed uncontrolled cases.*

*^c^Inactive: remission or controlled disease.*

*^d^The variable of Prednisolone dosage at vaccination had obvious colinearity with Bullous disease status at vaccination.*

*^e^Sinopharm, BBIBP-CorV; AstraZeneca, ChAdOx1 nCoV-19; COVIran Barekat, BIV1-CovIran. AIBDs, autoimmune bullous diseases; RTX, rituximab. The bold values indicate the significant p-value of <0.05.*

## Discussion

The current study assessed the effect of SARS-CoV-2 vaccination on AIBDs’ course in a large cohort of patients. Previous reports suggested exacerbation of AIBDs after tetanus and influenza vaccination (14–16). A similar phenomenon has been observed with regard to SARS-CoV-2 vaccine (6, 17). In a recent study, five patients with confirmed diagnosis of AIBDs were reported to develop disease exacerbation after the first COVID-19 vaccine. All patients had received mRNA vaccines at the remission phase of the disease, and only one experienced flare following the second dose (6). In another report, a patient with pemphigus vulgaris at remission on maintenance therapy with 5 mg/day of prednisone for 10 months presented new lesions after both doses of BNT162b2 vaccine. The exacerbations occurred 5 days after vaccine injections and were confirmed using histological and serological evaluation (17). Given the need for vaccination in AIBD patients, it is very important to have an estimate of post-vaccination disease exacerbation in a large population of patients.

Our results showed that only less than one-fifth of the AIBD patients experienced disease exacerbation post-SARS-CoV-2 vaccination. Moreover, there is no sufficient evidence to relate all the reported exacerbations to the vaccine with certainty, and some might be coincidental events. Therefore, while dermatologists encourage patients to be vaccinated against COVID-19, they should be aware of this possible event and inform their patients about it. It is noteworthy that exacerbation of the disease after the first dose does not preclude the administration of a second dose. According to our study, only a limited number of patients suffered from disease exacerbation after both vaccine shots, which is consistent with a previous report in this regard (6).

In addition to disease exacerbation, we detected five cases (three bullous pemphigoid and two pemphigus vulgaris) with a new diagnosis after vaccination. Numerous studies have reported similar cases induced by SARS-CoV-2 vaccination (7, 18–20). A recent review summarized demographic, clinical, and immunological characteristics of 35 case reports with new diagnoses following the COVID-19 vaccine. Of them, 26 were bullous pemphigoid, 6 pemphigus vulgaris, 2 linear IgA disease, and one pemphigus foliaceus. Contrary to the common epidemiology of AIBDs, they reported a female to male ratio of 1:1.7. The bullous lesions were presented after a median of 7 days (IQR: 3–14) following vaccine shots (21). In the largest case series of patients with SARS-CoV-2 vaccine-associated bullous pemphigoid, characteristics of 21 patients were compared to idiopathic disease. They reported similar clinical presentations, with a predominance of male patients. Immunopathological features were typical, but anti-BP230 autoantibody was remarkably reduced (22). It should be noted that some authors argue against the casualty relation between the SARS-CoV-2 vaccine and AIBDs as they recorded no increase in disease incidence in the year of vaccination (23).

The mechanisms triggering disease activity after vaccination are unclear, although both dysregulation of the immune system and molecular mimicry of vaccine adjuvants or antigens were previously suggested to play role in this regard. A recently published study has argued against the cross-reactivity relation between SARS-CoV-2 immunization and AIBDs. The authors in that study found that none of the 12 individuals with recent COVID-19 infection nor 12 individuals with SARS-CoV-2 immunization had any concomitant autoantibody of pemphigus or pemphigoid (24). Therefore, the role of the immune response following the SARS-CoV-2 vaccine would be highlighted. It is supposed that excessive generation of type I interferons and proinflammatory cytokine following vaccination would induce innate and adaptive immune cells proliferation (25). Triggering humoral immunity by regulatory T cells dysfunction in susceptible persons may contribute to autoantibody production in pemphigus and pemphigoid disorders (26). Cytotoxic T cell activation might also stimulate post-vaccination exacerbation of disease especially in pemphigoid group (27, 28). In addition, it is shown that SARS-CoV-2 vaccine is associated with complement dysregulation, which could be another reason for exacerbation of bullous pemphigoid and mucous membrane pemphigoid (29, 30).

In this study, patients who were vaccinated in the active phase of the disease were more prone to experience post-vaccine disease exacerbation with a number needed to harm of 3. Furthermore, disease exacerbation rate increased with a longer duration between the last rituximab infusion and vaccination. These findings can be important in the routine practice of dermatologists caring for patients with AIBDs, implying that it would be best to prescribe the COVID-19 vaccine when the patients are in the remission/controlled phase of the disease.

It has been shown that immunosuppressive drugs reduce the efficacy of SARS-CoV2 vaccines (31). Patients with immune-mediated skin conditions treated with immunosuppressants had low levels of serum anti-SARS-CoV-2 IgG antibodies, according to a recent study (32). The higher doses of corticosteroids in patients with active disease would attenuate the response to the vaccine. Therefore, vaccine administration in the remission/controlled phase of the disease would improve vaccine-induced immunity along with lowering the risk of disease exacerbation. This was previously suggested in the expert recommendations for the management of AIBDs during the COVID-19 pandemic, which can now be relied on with greater certainty (33). With regard to rituximab, the same group of experts suggested that vaccination should be completed more than 4 weeks prior to rituximab or 12–20 weeks after the last infusion. Although vaccination was found safe, vaccine efficacy, CD20-B cell count, and seroconversion rate were not studied in the present cohort; therefore, we cannot draw any conclusion regarding the optimal timing for obtaining the best efficacy.

The authors acknowledge the limitations of this study. It is important to mention that there might be multiple factors affecting AIBDs exacerbation. So, merely observing an exacerbation of the disease after vaccination cannot be concrete evidence for a probable association. Since the beginning of the COVID-19 pandemic, countries have been affected differently, and each has chosen a particular approach for prevention, vaccination, and treatment. Preexistence differences between countries may lead to variable results. Therefore, replicating the current study in further research from other regions would be crucial to reinforce the results.

Taking the following measures could further determine the effect of the SARS-CoV2 vaccine on disease exacerbation. First, performing laboratory assessments may help to elucidate the immunopathogenesis of this phenomenon, including different aspects of innate and adaptive immunity. Moreover, it could help detect patients who experience only serologic changes and minimal or no clinical findings after SARS-CoV2 vaccination. Second, a comparison of the exacerbation rates in vaccinated and unvaccinated patients during the same period with controlling other confounding factors would provide further information regarding the impact of SARS-CoV2 vaccines on the natural history of AIBDs. Despite these limitations, the message of this study regarding the low rate of exacerbation after SARS-CoV2 vaccines and identifying more susceptible individuals for this phenomenon can still be trusted.

## Conclusion

Together, these findings suggest that there might be an association between SARS-CoV-2 vaccination and exacerbation of AIBDs. Active disease was especially concerning with a number needed to harm of 3. Still, the benefits of vaccination undoubtedly outweigh the potential risk of complications. Vaccination is therefore strictly recommended for patients with AIBDs, although preferably in the remission/controlled phase of the disease.

## Data Availability Statement

The original contributions presented in this study are included in the article/Supplementary Material, further inquiries can be directed to the corresponding author.

## Ethics Statement

The studies involving human participants were approved by the Tehran University of Medical Sciences Ethics Committee (IR.TUMS.MEDICINE.REC.1400.911). Written informed consent for participation was not required for this study in accordance with the national legislation and the institutional requirements.

## Author Contributions

MD, HM, and KB contributed to conception and design of the study. NK, SD, and AS gathered data. NK and SD performed the statistical analysis and wrote the first draft of the manuscript. All authors contributed to manuscript revision, read, and approved the submitted version.

## Conflict of Interest

The authors declare that the research was conducted in the absence of any commercial or financial relationships that could be construed as a potential conflict of interest.

## Publisher’s Note

All claims expressed in this article are solely those of the authors and do not necessarily represent those of their affiliated organizations, or those of the publisher, the editors and the reviewers. Any product that may be evaluated in this article, or claim that may be made by its manufacturer, is not guaranteed or endorsed by the publisher.

## Supplementary Material

Supplementary Figure 1 The administrating rates of the first dose of SARS-CoV-2 vaccine in the study population and country’s general population.

## References

[1] Kasperkiewicz M Woodley DT. Association between vaccination and autoimmune bullous diseases: a systematic review. *J Am Acad Dermatol.* (2021) 86:1160–4. 10.1016/j.jaad.2021.04.061 33905786

[2] World Health Organization. *Use of Chest Imaging in COVID-19: A Rapid Advice Guide, 11 June 2020.* Geneva: World Health Organization (2020).

[3] Mahmoudi H Farid AS Nili A Dayani D Tavakolpour S Soori T Characteristics and outcomes of COVID-19 in patients with autoimmune bullous diseases: a retrospective cohort study. *J Am Acad Dermatol.* (2021) 84:1098–100. 10.1016/j.jaad.2020.12.043 33359593 PMC7836213

[4] Drenovska K Vassileva S Tanev I Joly P. Impact of COVID-19 on autoimmune blistering diseases. *Clin Dermatol.* (2021) 39:359–68. 10.1016/j.clindermatol.2021.01.007 34517993 PMC7955939

[5] Shakshouk H Daneshpazhooh M Murrell DF Lehman JS. Treatment considerations for patients with pemphigus during the COVID-19 pandemic. *J Am Acad Dermatol.* (2020) 82:e235–6. 10.1016/j.jaad.2020.04.005 32283243 PMC7146668

[6] Damiani G Pacifico A Pelloni F Iorizzo M. The first dose of COVID-19 vaccine may trigger pemphigus and bullous pemphigoid flares: is the second dose therefore contraindicated? *J Eur Acad Dermatol Venereol.* (2021) 35:e645–7. 10.1111/jdv.17472 34169578 PMC8447358

[7] Solimani F Mansour Y Didona D Dilling A Ghoreschi K Meier K. Development of severe pemphigus vulgaris following SARS-CoV-2 vaccination with BNT162b2. *J Eur Acad Dermatol Venereol.* (2021) 35:e649–51. 10.1111/jdv.17480 34169588 PMC8447452

[8] Hatami P Balighi K Nicknam Asl H Aryanian Z. COVID vaccination in patients under treatment with rituximab: a presentation of two cases from Iran and a review of the current knowledge with a specific focus on pemphigus. *Dermatol Therapy.* (2022) 35:e15216. 10.1111/dth.15216 34811862 PMC9011959

[9] Olson N Eckhardt D Delano A. New-onset bullous pemphigoid in a COVID-19 patient. *Case Rep Dermatol Med.* (2021) 2021:5575111. 10.1155/2021/5575111 34211788 PMC8187076

[10] Mahmoudi H Balighi K Tavakolpour S Daneshpazhooh M. Unexpected worsening of pemphigus vulgaris after rituximab: a report of three cases. *Int Immunopharmacol.* (2019) 71:40–2. 10.1016/j.intimp.2019.02.037 30877872

[11] Murrell DF Peña S Joly P Marinovic B Hashimoto T Diaz LA Diagnosis and management of pemphigus: recommendations of an international panel of experts. *J Am Acad Dermatol.* (2020) 82:575.e–85.e. 10.1016/j.jaad.2018.02.021 29438767 PMC7313440

[12] Chams-Davatchi C Esmaili N Daneshpazhooh M Valikhani M Balighi K Hallaji Z Randomized controlled open-label trial of four treatment regimens for pemphigus vulgaris. *J Am Acad Dermatol.* (2007) 57:622–8. 10.1016/j.jaad.2007.05.024 17583373

[13] Barbhaiya M Levine JM Bykerk VP Jannat-Khah D Mandl LA. Systemic rheumatic disease flares after SARS-CoV-2 vaccination among rheumatology outpatients in New York City. *Ann Rheumat Dis.* (2021) 80:1352–4. 10.1136/annrheumdis-2021-220732 34158370

[14] Makhneva NV Davidenko EB Zenkevich EV Beletskaya LV Mahneva NV Davydenko EB Bullous pemphigoid exacerbation after vaccination. *RJSKD.* (2011) 14:14–7. 10.17816/dv36515

[15] De Simone C Caldarola G D’Agostino M Zampetti A Amerio P Feliciani C. Exacerbation of pemphigus after influenza vaccination. *Clin Exp Dermatol.* (2008) 33:718–20. 10.1111/j.1365-2230.2008.02835.x 18681883

[16] Korang K Ghohestani R Krieg T Uitto J Hunzelmann N. Exacerbation of pemphigus foliaceus after tetanus vaccination accompanied by synthesis of auto-antibodies against paraneoplastic pemphigus antigens. *Acta Dermato Venereol.* (2002) 82:482–3. 10.1080/000155502762064755 12575868

[17] Avallone G Giordano S Astrua C Merli M Senetta R Conforti C Reply to ‘The first dose of COVID-19 vaccine may trigger pemphigus and bullous pemphigoid flares: is the second dose therefore contraindicated?’ by Damiani G et al. *J Eur Acad Dermatol Venereol.* (2022) 36:e433–5. 10.1111/jdv.17959 35067994

[18] Saffarian Z Samii R Ghanadan A Vahidnezhad H. De novo severe pemphigus vulgaris following SARS-CoV-2 vaccination with BBIBP-CorV. *Dermatol Therapy.* (2022) 35:e15448. 10.1111/dth.15448 35289040 PMC9111647

[19] Young J Mercieca L Ceci M Pisani D Betts A Boffa MJ. A case of bullous pemphigoid after the SARS-CoV-2 mRNA vaccine. *J Eur Acad Dermatol Venereol JEADV.* (2022) 36:e13–6. 10.1111/jdv.17676 34547137 PMC8661451

[20] Gambichler T Hamdani N Budde H Sieme M Skrygan M Scholl L Bullous pemphigoid after SARS-CoV-2 vaccination: spike-protein-directed immunofluorescence confocal microscopy and T-cell-receptor studies. *Br J Dermatol.* (2022) 186:728–31. 10.1111/bjd.20890 34773638 PMC8653321

[21] Calabria E Canfora F Mascolo M Varricchio S Mignogna MD Adamo D. Autoimmune mucocutaneous blistering diseases after SARS-Cov-2 vaccination: a Case report of Pemphigus Vulgaris and a literature review. *Pathol Res Pract.* (2022) 232:153834. 10.1016/j.prp.2022.153834 35278817 PMC8896864

[22] Maronese CA Caproni M Moltrasio C Genovese G Vezzoli P Sena P Bullous pemphigoid associated With COVID-19 vaccines: an Italian multicentre study. *Front Med.* (2022) 9:841506. 10.3389/fmed.2022.841506 35295599 PMC8918943

[23] Russo R Gasparini G Cozzani E D’Agostino F Parodi A. Absolving COVID-19 vaccination of autoimmune bullous disease onset. *Front Immunol.* (2022) 13:834316. 10.3389/fimmu.2022.834316 35251024 PMC8895245

[24] Kasperkiewicz M Bednarek M Tukaj S. Case report: circulating Anti-SARS-CoV-2 antibodies do not cross-react with pemphigus or pemphigoid autoantigens. *Front Med.* (2021) 8:807711. 10.3389/fmed.2021.807711 34988105 PMC8720918

[25] Sadarangani M Marchant A Kollmann TR. Immunological mechanisms of vaccine-induced protection against COVID-19 in humans. *Nat Rev Immunol.* (2021) 21:475–84. 10.1038/s41577-021-00578-z 34211186 PMC8246128

[26] Wack S Patton T Ferris LK. COVID-19 vaccine safety and efficacy in patients with immune-mediated inflammatory disease: review of available evidence. *J Am Acad Dermatol.* (2021) 85:1274–84. 10.1016/j.jaad.2021.07.054 34363909 PMC8336973

[27] Chow S Rizzo C Ravitskiy L Sinha AA. The role of T cells in cutaneous autoimmune disease. *Autoimmunity.* (2005) 38:303–17. 10.1080/08916930500124429 16206513

[28] Fang H Li Q Wang G. The role of T cells in pemphigus vulgaris and bullous pemphigoid. *Autoimmun Rev.* (2020) 19:102661. 10.1016/j.autrev.2020.102661 32942041

[29] Portuguese AJ Sunga C Kruse-Jarres R Gernsheimer T Abkowitz J. Autoimmune- and complement-mediated hematologic condition recrudescence following SARS-CoV-2 vaccination. *Blood Adv.* (2021) 5:2794–8. 10.1182/bloodadvances.2021004957 34255033 PMC8276576

[30] Edwards G Diercks GFH Seelen MAJ Horvath B van Doorn MBA Damman J. Complement activation in autoimmune bullous dermatoses: a comprehensive review. *Front Immunol.* (2019) 10:1477. 10.3389/fimmu.2019.01477 31293600 PMC6606728

[31] Deepak P Kim W Paley MA Yang M Carvidi AB Demissie EG Effect of immunosuppression on the immunogenicity of mRNA vaccines to SARS-CoV-2 : a prospective cohort study. *Ann Internal Med.* (2021) 174:1572–85. 10.7326/m21-1757 34461029 PMC8407518

[32] Seree-aphinan C Chanprapaph K Rattanakaemakorn P Setthaudom C Suangtamai T Pomsoong C Inactivated COVID-19 vaccine induces a low humoral immune response in a subset of dermatological patients receiving immunosuppressants. *Front Med.* (2021) 8:769845. 10.3389/fmed.2021.769845 34957149 PMC8692273

[33] Kasperkiewicz M Schmidt E Amagai M Fairley JA Joly P Murrell DF Updated international expert recommendations for the management of autoimmune bullous diseases during the COVID-19 pandemic. *JEADV.* (2021) 35:e412–4. 10.1111/jdv.17207 33655539 PMC8013840

