# Supplementary figures and images for: Exacerbation of Autoimmune Bullous Diseases After Severe Acute Respiratory Syndrome Coronavirus 2 Vaccination: Is There Any Association?
Source: Front Med (Lausanne). 2022 Jul 19;9:957169. doi: 10.3389/fmed.2022.957169 (PMC9344059; doi:10.3389/fmed.2022.957169)

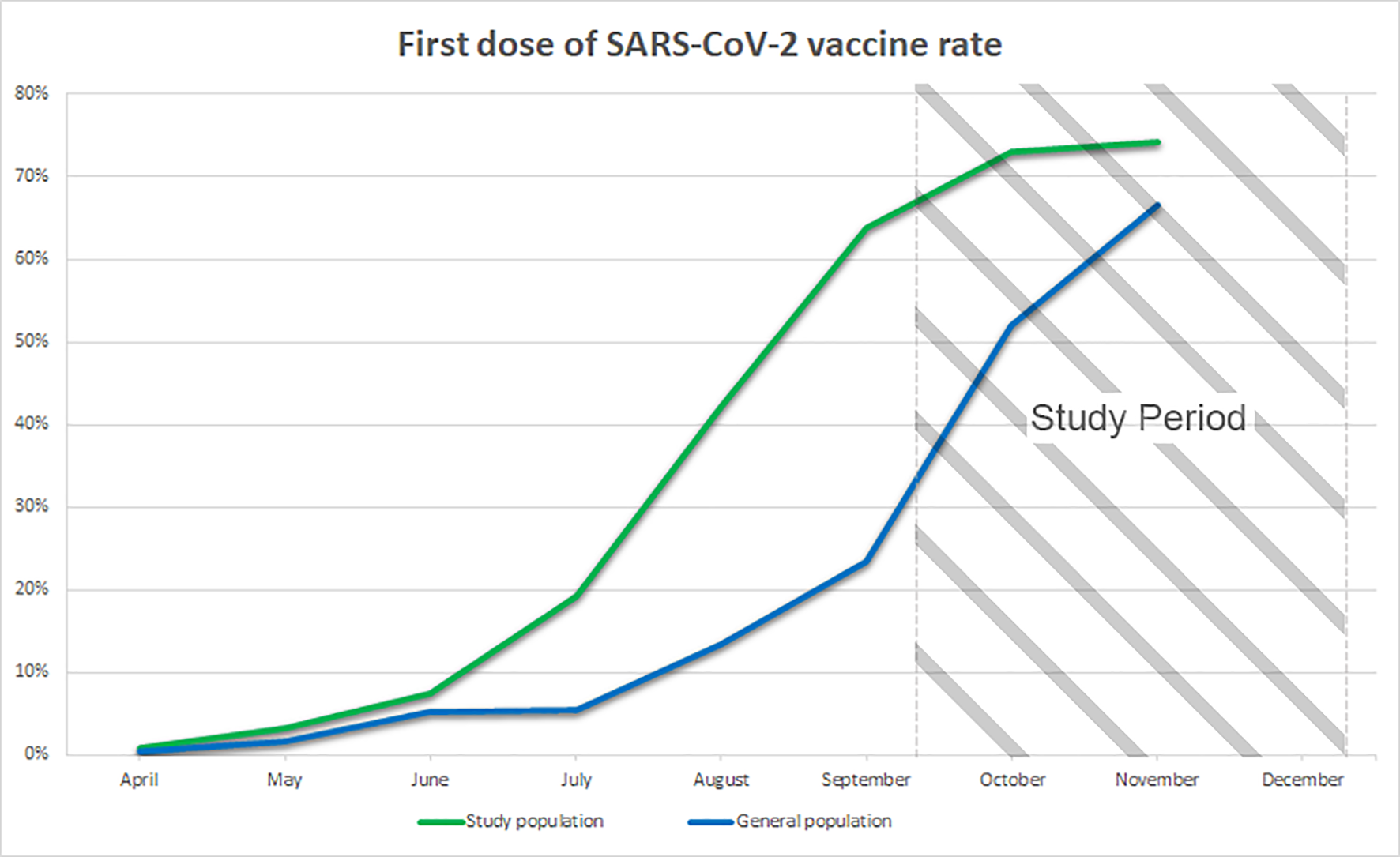

Supplement: Supplementary Figure 1 — The administrating rates of the first dose of SARS-CoV-2 vaccine in the study population and country’s general population. [file Image_1.JPEG]
