# Supplementary material for: Exacerbation of Autoimmune Bullous Diseases After Severe Acute Respiratory Syndrome Coronavirus 2 Vaccination: Is There Any Association?
Source: Front Med (Lausanne). 2022 Jul 19;9:957169. doi: 10.3389/fmed.2022.957169 (PMC9344059; doi:10.3389/fmed.2022.957169)
Supplement: Supplementary file 2 [file Table_1.docx]

**Supplementary** **Table 1** Detailed information of disease exacerbation in different autoimmune bullous diseases subtypes

| Type of AIBDs |  |  |  |
| --- | --- | --- | --- |
| Pemphigus vulgaris )n=361( |  |  |  |
| Disease duration until vaccination, months, median (IQR) |  | 59.8 (33.4-109.5) |  |
| Bullous disease status at vaccination, n (%) | CR on minimal or off-therapy | 183 (50.7%) |  |
|  | PR on minimal or off-therapy | 127 (35.2%) |  |
|  | Controlled | 19 (5.3%) |  |
|  | Relapse | 25 (6.9%) |  |
|  | Uncontrolled new cases | 5 (1.4%) |  |
|  | Undiagnosed or healthy | 2 (0.6%) |  |
| Disease severity at vaccination, median (IQR) | PDAI score | 0 (0-1) |  |
| Medication at vaccination, n (%) | Prednisolone, mg (dose, median [IQR]) | 273 (75.6%), (5, [3.7-7.5]) |  |
|  | Mycophenolate mofetil | 12 (3.3%) |  |
|  | Topical | 22 (6.1%) |  |
|  | Other systemic | 9 (2.5%) |  |
|  | No medication | 85 (23.5%) |  |
| Vaccination |  | First dose vaccination | Second dose vaccination |
|  | Sinopharm | 309 (85.6%) | 284 (78.7%) |
|  | AstraZeneca | 29 (8.0%) | 23 (6.4%) |
|  | COVIran Barekat | 16 (4.4%) | 14 (3.9%) |
|  | Other | 7 (1.9%) | 7 (2.1%) |
| Disease exacerbation after vaccination, n (%) | Disease worsening | 10 (2.7%) | 19 (5.8%) |
|  | Minor relapse | 17 (4.7%) | 12 (3.7%) |
|  | Major relapse | 9 (2.5%) | 5 (1.5%) |
|  | Total | 36 (9.9%) | 37 (11.3%) |
| New diagnosis pemphigus vulgaris after vaccination, n (%) |  | 2 (0.6%) | _ |
| Interval from vaccination to disease exacerbation (days), median (IQR) |  | 7 (4.2-13.5) | 7 (3.2-10) |
| Medication taken for post-vaccination disease activity, n (%) | No altered medication | 4 (10.5%) | 4 (10.8%) |
|  | Topical | 5 (13.2%) | 4 (10.8%) |
|  | Increase medication dosage | 30 (78.9%) | 29 (78.4%) |
|  | New medication (other than RTX) | 3 (7.9%) | 1 (2.7%) |
|  | RTX | 5 (13.2%) | 15 (40.5%) |
| Disease exacerbation rate according to disease status, n (%) | Active | 15/30 (50%) |  |
|  | Remission/Controlled | 43/329 (13.1%) |  |
|  | Total | 58/359 (16.1%) |  |
| Pemphigus foliaceus )n=38( |  |  |  |
| Disease duration until vaccination, months, median (IQR) |  | 62.8 (31.4-137.5) |  |
| Bullous disease status at vaccination, n (%) | CR on minimal or off-therapy | 15 (39.5%) |  |
|  | PR on minimal or off-therapy | 18 (47.4%) |  |
|  | Controlled | 0 (0%) |  |
|  | Relapse | 1 (2.6%) |  |
|  | Uncontrolled new cases | 4 (10.5%) |  |
| Disease severity at vaccination, median (IQR) | PDAI score | 0 (0-3) |  |
| Medication at vaccination, n (%) | Prednisolone, mg (dose, median [IQR]) | 26 (68.4%), (5, [2.5-5]) |  |
|  | Mycophenolate mofetil | 1 (2.6%) |  |
|  | Topical | 7 (18.4%) |  |
|  | Other systemic | 3 (7.9%) |  |
|  | No medication | 10 (26.3%) |  |
| Vaccination |  | First dose vaccination | Second dose vaccination |
|  | Sinopharm | 29 (76.3%) | 27 (71.1%) |
|  | AstraZeneca | 3 (7.9%) | 2 (5.3%) |
|  | COVIran Barekat | 5 (13.2%) | 5 (13.2%) |
|  | Other | 1 (2.6%) | 1 (2.6%) |
| Disease exacerbation after vaccination, n (%) | Disease worsening | 2 (5.3%) | 2 (5.7%) |
|  | Minor relapse | 2 (5.3%) | 0 (2.8%) |
|  | Major relapse | 0 (0%) | 0 (0%) |
|  | Total | 4 (10.5%) | 2 (5.7%) |
| Interval from vaccination to disease exacerbation (days), median (IQR) |  | 10 (3.2-13) | 6.5 (3-10) |
| Medication taken for post-vaccination disease activity, n (%) | No altered medication | 2 (50.0%) | 0 (0%) |
|  | Topical | 2 (50.0%) | 0 (0%) |
|  | Increase medication dosage | 2 (50.0%) | 2 (100.0%) |
|  | New medication (other than RTX) | 0 (0%) | 0 (0%) |
|  | RTX | 0 (0%) | 1 (50.0%) |
| Disease exacerbation rate according to disease status, n (%) | Active | 2/5 (40%) |  |
|  | Remission/Controlled | 2/33 (6.0%) |  |
|  | Total | 4/38 (10.5%) |  |
| Bullous pemphigoid (n=29) |  |  |  |
| Disease duration until vaccination, months, median (IQR) |  | 23.5 (12.1-37.5) |  |
| Bullous disease status at vaccination, n (%) | CR on minimal or off-therapy | 15 (51.7%) |  |
|  | PR on minimal or off-therapy | 7 (24.1%) |  |
|  | Controlled | 1 (3.4%) |  |
|  | Relapse | 2 (6.9%) |  |
|  | Uncontrolled new cases | 1 (3.4%) |  |
|  | Undiagnosed or healthy | 3 (10.3%) |  |
| Disease severity at vaccination, median (IQR) | BPDAI score | 0 (0-3) |  |
| Medication at vaccination, n (%) | Prednisolone, mg (dose, median [IQR]) | 13 (44.8%), (7.5, [3.75-10]) |  |
|  | Mycophenolate mofetil | 1 (3.4%) |  |
|  | Topical | 8 (27.6%) |  |
|  | Other systemic | 1 (3.4%) |  |
|  | No medication | 14 (48.2%) |  |
| Vaccination |  | First dose vaccination | Second dose vaccination |
|  | Sinopharm | 24 (82.8%) | 19 (65.5%) |
|  | AstraZeneca | 4 (13.8%) | 3 (10.3%) |
|  | COVIran Barekat | 1 (3.4%) | 1 (3.4%) |
|  | Other | 0 (0%) | 0 (0%) |
| Disease exacerbation after vaccination, n (%) | Disease worsening | 0 (0%) | 0 (0%) |
|  | Minor relapse | 1 (3.4%) | 0 (0%) |
|  | Major relapse | 0 (0%) | 0 (0%) |
|  | Total | 1 (3.4%) | 0 (0%) |
| New diagnosis bullous pemphigoid after vaccination, n (%) |  | 1 (3.4%) | 2 (8.7%) |
| Interval from vaccination to disease exacerbation (days), median (IQR) |  | 2 | - |
| Medication taken for post-vaccination disease activity, n (%) | No altered medication | 0 (0%) | 0 (0%) |
|  | Topical | 1 (50.0%) | 1 (50.0%) |
|  | Increase medication dosage | 0 (0%) | 0 (0%) |
|  | New medication (other than RTX) | 1 (50.0%) | 1 (50.0%) |
|  | RTX | 0 (0%) | 0 (0%) |
| Disease exacerbation rate according to disease status, n (%) | Active | 0/3 (0%) |  |
|  | Remission/Controlled | 1/23 (4.3%) |  |
|  | Total | 3/26 (3.8%) |  |
| Mucous membrane pemphigoid (n=13) |  |  |  |
| Disease duration until vaccination, months, median (IQR) |  | 58.3 (30.3-105.4) |  |
| Bullous disease status at vaccination, n (%) | CR on minimal or off-therapy | 5 (38.5%) |  |
|  | PR on minimal or off-therapy | 7 (53.9%) |  |
|  | Controlled | 0 (0%) |  |
|  | Relapse | 1 (7.7%) |  |
|  | Uncontrolled new cases | 0 (0%) |  |
| Disease severity at vaccination, median (IQR) | MMPDAI score | 0 (0-2) |  |
| Medication at vaccination, n (%) | Prednisolone, mg (dose, median [IQR]) | 9 (69.2%), (7.5, [5-12.5]) |  |
|  | Mycophenolate mofetil | 3 (23.1%) |  |
|  | Topical | 3 (23.1%) |  |
|  | Other systemic | 3 (23.1%) |  |
|  | No medication | 3 (23.1%) |  |
| Vaccination |  | First dose vaccination | Second dose vaccination |
|  | Sinopharm | 12 (92.3%) | 13 (100%) |
|  | AstraZeneca | 0 (0%) | 0 (0%) |
|  | COVIran Barekat | 0 (0%) | 0 (0%) |
|  | Other | 1 (7.7%) | 0 (0%) |
| Disease exacerbation after vaccination, n (%) | Disease worsening | 0 (0%) | 2 (15.4%) |
|  | Minor relapse | 1 (7.7%) | 1 (7.7%) |
|  | Major relapse | 0 (0%) | 0 (0%) |
|  | Total | 1 (7.7%) | 3 (23.1%) |
| Interval from vaccination to disease exacerbation (days), median (IQR) |  | 1 | 7 (1-7) |
| Medication taken for post-vaccination disease activity, n (%) | No altered medication | 0 (0%) | 0 (0%) |
|  | Topical | 0 (0%) | 0 (0%) |
|  | Increase medication dosage | 1(100%) | 3 (100%) |
|  | New medication (other than RTX) | 0 (0%) | 0 (0%) |
|  | RTX | 0 (0%) | 2 (66.7%) |
| Disease exacerbation rate according to disease status, n (%) | Active | 1/1 (100%) |  |
|  | Remission/Controlled | 2/12 (16.7%) |  |
|  | Total | 3/13 (23.1%) |  |
| Linear IgA disease (n=2) |  |  |  |
| Disease duration until vaccination, months |  | (24, 38) |  |
| Bullous disease status at vaccination, n (%) | PR on minimal or off-therapy | 2 (100%) |  |
| Disease severity at vaccination, median (IQR) | BPDAI score | 1.5 (0-3) |  |
| Medication at vaccination, n (%) | Prednisolone, mg (dose) | 2 (100%), (10, 10) |  |
|  | Other systemic | 2 (100%) |  |
| Vaccination |  | First dose vaccination | Second dose vaccination |
|  | Sinopharm | 2 (100%) | 2 (100%) |
| Disease exacerbation after vaccination, n (%) | Total | 0 (0%) | 0 (0%) |
| Disease exacerbation rate according to disease status, n (%) | Active | - |  |
|  | Remission/Controlled | 0/2 (0%) |  |
|  | Total | 0/2 (0%) |  |
| Epidermolysis bullosa acquisita (n=2) |  |  |  |
| Disease duration until vaccination, months |  | 6, 76 |  |
| Bullous disease status at vaccination, n (%) | CR on minimal or off-therapy | 1 (50%) |  |
|  | Controlled | 1 (50%) |  |
| Disease severity at vaccination | MMPDAI score | 0, 3 |  |
| Medication at vaccination, n (%) | Prednisolone, mg (dose) | 2 (100%), (10, 15) |  |
| Vaccination | Sinopharm | 2 (100%) | 2 (100%) |
| Disease exacerbation after vaccination, n (%) | Total | 0 (0%) | 0 (0%) |
| Disease exacerbation rate according to disease status, n (%) | Active | - |  |
|  | Remission/Controlled | 0/2 (0%) |  |
|  | Total | 0/2 (0%) |  |
| Paraneoplastic pemphigus (n=1) |  |  |  |
| Disease duration until vaccination, months |  | 3.5 |  |
| Bullous disease status at vaccination, n (%) | Uncontrolled new cases | 1 (100%) |  |
| Disease severity at vaccination | PDAI score | 6 |  |
| Medication at vaccination, n (%) | Prednisolone, mg (dose, median [IQR]) | 1 (100%), (30) |  |
| Vaccination |  | First dose vaccination | Second dose vaccination |
|  | Sinopharm | 1 (100%) | 1 (100%) |
| Disease exacerbation after vaccination, n (%) | Total | 0 (0%) | 0 (0%) |
| Disease exacerbation rate according to disease status, n (%) | Active | 0/1 (0%) |  |
|  | Remission/Controlled | - |  |
|  | Total | 0/1 (0%) |  |

Abbreviations: AIBDs, autoimmune bullous diseases; SD, standard deviation; IQR, interquartile range; CR, complete remission; PR, partial remission; PDAI, Pemphigus Disease Area Index; RTX, rituximab, BPDAI, Bullous Pemphigoid Disease Area Index; MMPDAI, Mucous Membrane Pemphigoid Disease Area Index
